# High Power-Conversion Efficiency of Lead-Free Perovskite Solar Cells: A Theoretical Investigation

**DOI:** 10.3390/mi13122201

**Published:** 2022-12-12

**Authors:** Ahmad Umar, Sadanand, Pravin Kumar Singh, D. K. Dwivedi, Hassan Algadi, Ahmed A. Ibrahim, Mohsen A. M. Alhammai, Sotirios Baskoutas

**Affiliations:** 1 Department of Chemistry, Faculty of Science and Arts, and Promising Centre for Sensors and Electronic Devices (PCSED), Najran University, Najran 11001, Saudi Arabia; 2 Department of Materials Science and Engineering, The Ohio State University, Columbus, OH 43210, USA; 3 Department of Applied Sciences, Galgotias College of Engineering and Technology, Greater Noida 201306, India; 4 Institute of Advanced Materials, IAAM, Gammalkilsvägen 18, 590 53 Ulrika, Sweden; 5 Photonics and Photovoltaic Research Lab, Department of Physics and Material Science, Madan Mohan Malaviya University of Technology, Gorakhpur 273010, India; 6 Department of Electrical Engineering, College of Engineering, Najran University, Najran 11001, Saudi Arabia; 7 Department of Materials Science, University of Patras, 265 04 Patras, Greece

**Keywords:** perovskite, electron transport layer, hole transport layer, SCAPS-1D

## Abstract

Solar cells based on lead-free perovskite have demonstrated great potential for next-generation renewable energy. The SCAPS-1D simulation software was used in this study to perform novel device modelling of a lead-free perovskite solar cell of the architecture ITO/WS_2_/CH_3_NH_3_SnI_3_/P3HT/Au. For the performance evaluation, an optimization process of the different parameters such as thickness, bandgap, doping concentration, etc., was conducted. Extensive optimization of the thickness and doping density of the absorber and electron transport layer resulted in a maximum power-conversion efficiency of 33.46% for our designed solar cell. Because of the short diffusion length and higher defect density in thicker perovskite, an absorber thickness of 1.2 µm is recommended for optimal solar cell performance. Therefore, we expect that our findings will pave the way for the development of lead-free and highly effective perovskite solar cells.

## 1. Introduction

A photovoltaic solar cell is a promising renewable and non-polluted source of energy [1]. In terms of research and development, halide-based perovskite solar cells (PSCs) are the fastest growing photovoltaic technology [2,3,4]. The first potential report of halide-based perovskite solar cells appeared in 2009. In such studies, organic−inorganic (CH_3_NH_3_PbI_3_) hybrid perovskite has been used as a light-sensitizer in a dye-sensitized solar cell and a power conversion efficiency of 3.8% has been achieved. The ability of halide perovskites to operate not only as powerful light absorbers but also as efficient electron and hole conductors was demonstrated in the literature [5,6,7] with power conversion efficiencies of 10.9% and 9.7%, respectively. These findings led to the start of a global effort to improve perovskite cell efficiency beyond 20%. The Pb-based perovskite solar cells (PSCs) have gained a lot of potential over silicon-based solar cells due to their ease of fabrication and low cost. Lead-based halide perovskite also exhibits several desirable optoelectronic properties such as a high absorption coefficient, a comparatively moderate bandgap, and a high charge diffusion length [7,8,9].

Considering these great advances towards high performance, the toxicity and lack of stability of the absorber layer in lead-based PSCs remains a serious problem for their widespread commercialization [10,11,12]. One potential way to address these concerns is to replace the hazardous toxic lead in the PSC’s CH_3_NH_3_PbI_3_ absorber layer with a suitable non-toxic element. The methylammonium tin iodide CH_3_NH_3_SnI_3_ has, amongst others, become a viable alternative due to its eco-friendliness, as well as its smaller effective mass of holes [13], excellent mobility [14], narrower bandgap, and wider visible absorption spectrum [15].

The study of the characteristics of perovskite solar cell materials, as well as their control through accurate modelling, can lead to the production of efficient and cost-effective solar cells [16,17]. To build cost-effective, efficient, and lead-free PSCs, the electrical and optical properties of CH_3_NH_3_SnI_3_ must be explored using modelling before dealing with very complex fabrication processes. The main objective of this research is to design lead-free PSCs with an improved efficiency. In this study, we have used a solar cell capacitance simulator in one dimension (SCAPS-1D) program. To get the highest feasible PCE, different aspects were optimized. Initially, we have calibrated the device structure with previously reported experimental work [18]. Then, an investigation of the proposed device structure ITO/WS_2_/CH_3_NH_3_SnI_3_/P3HT/Au has been performed.

There has been a lot of interest in WS_2_ as ETL due to its potential as an electron transport layer in thin film solar cells [19]. It is easily available at a low cost and is less hazardous than other transition metal dichalcogenides (TMDC) compounds. The development of WS_2_ in thin film solar cells is still in its infancy compared with other photovoltaic materials [20]. Due to its superior optoelectronic properties, tungsten disulfide (WS_2_) has become the primary material for thin film solar cells. Its tunable bandgap is an essential feature that is usually ignored. WS_2_ has a large direct bandgap (>2 eV) and a small indirect bandgap (~1.3 eV) [20,21,22].

Furthermore, it exhibits high carrier mobility, good conductivity, native n-type semiconducting characteristics [23], and excellent electron conduction properties [24]. Moreover, it can be deposited through a solution process or by sputtering at low temperature [25]. Homo-polymer poly (3-hexylthiophene) (P3HT) as an HTL is one of the few viable choices for commercial Organic Photovoltaic (OPV). Its application in large-area, roll-to-roll printed solar cells has already been amply shown [26,27]. Additionally, the semi-crystalline structure of P3HT, in contrast to more amorphous polymers, is nearly unique in setting an acceptable morphological length-scale for bulk heterojunction OPV from a variety of solvents and processing conditions, as well as giving it outstanding charge transport properties [28,29]. P3HT has attracted great interest as a polymeric hole-selective material for perovskite solar cells due to its low cost [30], wide band-gap [31], relatively high hole mobility [32], high thermal stability [33], scalable solution processability [34], robust hydrophobicity, and oxygen impermeability [35].

An analysis of the impact of CH_3_NH_3_SnI_3_ as an absorber layer with different thicknesses and bandgap is performed. After this, the impact of ETL (WS_2_) with varying thicknesses and doping concentrations on the performance of PSCs has been investigated. At the end of this simulation analysis, the optimized device structure with the highest efficiency of 33.36% is obtained.

## 2. Device Structure and Simulation Methodology

**Figure 1:**
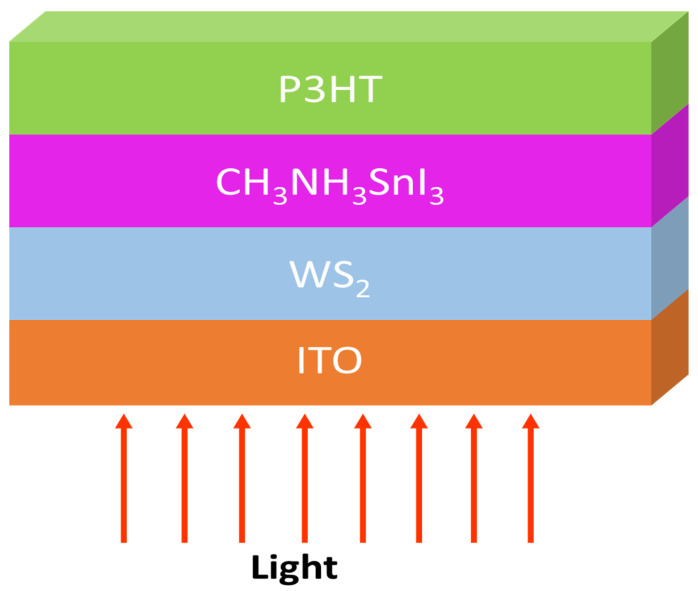
Schematic device structure of perovskite solar cell.

The perovskite solar cell device structure consists of Glass/ITO/WS_2_/CH_3_NH_3_SnI_3_/P3HT/Au, as shown in Figure 1. In this device design, ITO serves as an electron transport layer, which is covered by an N-type (WS_2_) material. The organic and inorganic intrinsic perovskite CH_3_NH_3_SnI_3_ serves as the absorber layer, while the p-type P3HT serves as the hole transporting layer on which the contact is formed.

The SCAPS-1D program was used to simulate a solar cell device. It is freely available to the research community. Solar radiation of AM_1.5 spectrum (1 kW/m^2^) is illuminated from the ITO window layer of the perovskite device structure. To start simulation one has to insert the material parameters by selecting the option ‘set problem’, click on ‘add the layer’, then insert the electrical and optical properties of the suitable material, such as thickness, electron affinity, dielectric permittivity (relative), bandgap, electron and hole thermal velocity (cm/s), electron and hole mobility (cm^2^/V-s), donor density N_D_ (cm^−3^), acceptor density N_A_ (cm^−3^). Then, insert the absorption coefficient (α) in the absorption model and recombination model, add the recombination details, and include the defects of the material [36,37]. Similarly, add the layers of the different materials and make the different types of solar cells. After adding the parameters as mentioned above, one has to check the illumination of light and whether or not light is passing through glass substrate (FTO, AZO, ITO, etc.) and through ETL layer which has less thickness (to allow the whole light to be absorbed in the absorber layer). Check whether the connections of the voltage are connected properly or not. Then, set the working point values to room temperature (300 K) and set the frequency. Add the series and shunt resistance values, ideally the series resistance would be low and the shunt resistance would be very high.

The SCAPS-1D simulation software is superior to other simulation software because it gives good consistency between experimental and simulated results. In the present simulation, series and shunt resistances are taken as 1 Ω and 10^6^ Ω  respectively. The effect of the dangling bond on the interface of the materials has been ignored. Benchmarking is achieved by matching the correct defect design before starting the simulation, therefore, the simulated outcome matches the corresponding experimentally tested values.

The parameters used in the simulation for the perovskite solar cell with device structure Glass/ITO/WS_2_/CH_3_NH_3_SnI_3_/P3HT/Au are listed in Table 1.

## 3. Results and Discussion

### 3.1. The Effect of the Thickness and Doping Concentration of the Absorber Layer

**Figure 2:**
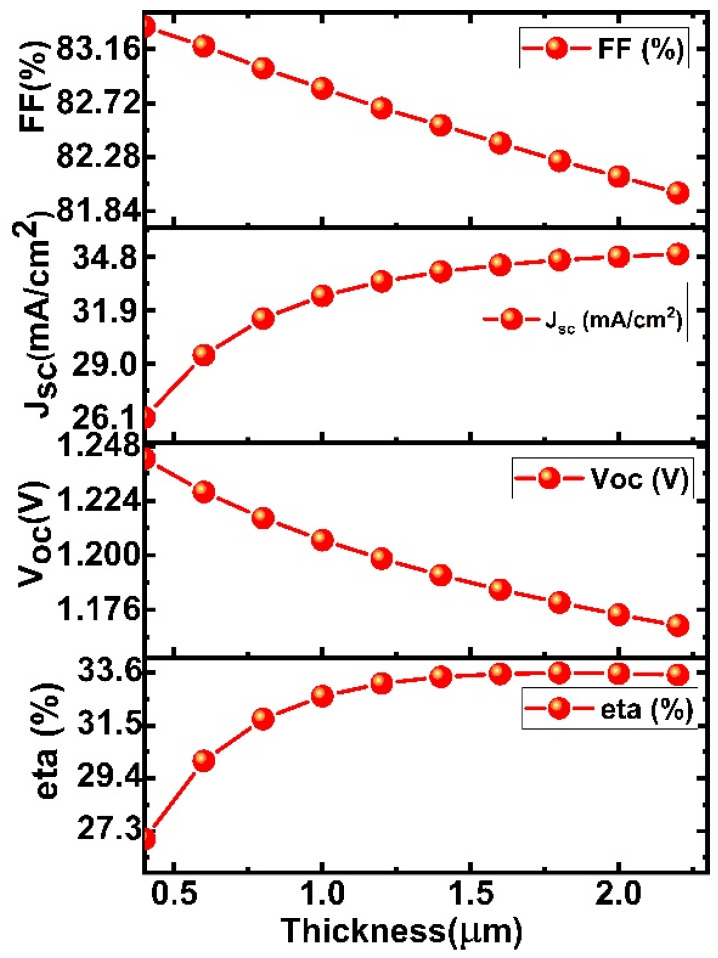
Effect of the perovskite layer thickness on solar cell parameters (J_sc_, V_oc_, FF, and η].

The impact of the active perovskite layer thickness on the efficiency of the solar device will be explored in this section. Absorber layers play a vital role in the improvement of the performance of the solar device [39,40]. All parameters, such as bandgap, thickness, and doping concertation, play an important role in optimizing performance. Figure 2 illustrates the variation of the electrical parameters with thickness, such as V_oc_ J_sc_ and FF, η. The simulated outcome shows that solar cell parameters are highly dependent on the thickness of the perovskite layer. The electrical parameters, such as J_sc_, and η, increase with the increase in the thickness of the perovskite layer while the FF and V_oc_ decrease with a further rise in thickness. When increasing the absorber layer thickness, it has been observed that the J_sc_, and eta performance improve but the V_oc_ performance degrades.

Since, as the absorber layer thickness increases, more photons are absorbed, and as photons penetrate deeper into the absorber layer, more electron-hole pairs are produced which increases the performance of the device [41]. The decrease in V_oc_ is caused by an increase in the dark saturation current, which enhances charge carrier recombination [42]. This is addressed by the photo-generated current and dark saturation current’s dependence on open-circuit voltage, which is expressed as [42,43]

(1) $$V_{\mathrm{oc}}=\frac{\mathrm{kT}}{q}\mathrm{Ln}\left[\frac{J_{\mathrm{sc}}}{J_{0}}+1\right]$$

Here J_sc_ represents the photo-generated current density, kT/q represents the thermal voltage, and J_0_ represents the saturation current density

**Figure 3:**
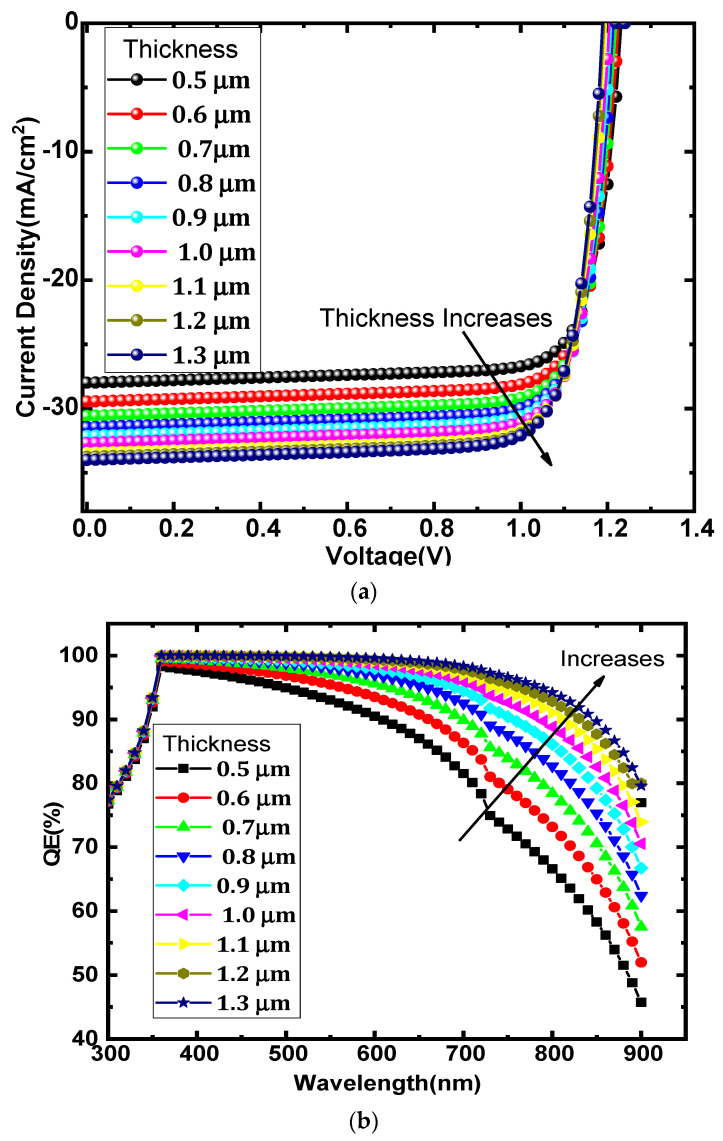
(**a**) Current density vs. voltage characteristics and (**b**) quantum efficiency vs. wavelength curve with varied thickness of the absorber layer.

The JV characteristics have been recorded by varying the thickness of the absorber layer, as shown in Figure 3a. Current density vs. voltage characteristics show an increase with increasing absorber layer thickness. As the thickness of the absorber layer increases, the area under the curve also enhances, which results in an increase in the Jsc values because more photons fall on it [40,41,42].

The external quantum efficiency spectra of the proposed device are shown in Figure 3b. The relations between quantum efficiency (QE) and wavelength curve show that the % QE increases with varying the absorber layer thickness. As the thickness of the absorber layer increases, more light is absorbed, resulting in a large number of carriers. These excess carriers led to the increase in the Jsc and PCE of the perovskite device [41,42]. Firstly, QE increases rapidly (up to 1.2 m) with the increase in the thickness of the absorber layer and after it gets saturated (as shown in Figure 3b). Considering the short diffusion length and higher defect density in thicker perovskite, a thickness of 1.2 µm for the absorber is appropriate for obtaining optimal solar cell performance.

### 3.2. Effect of Absorber’s Bandgap on Solar Cell Performance

**Figure 4:**
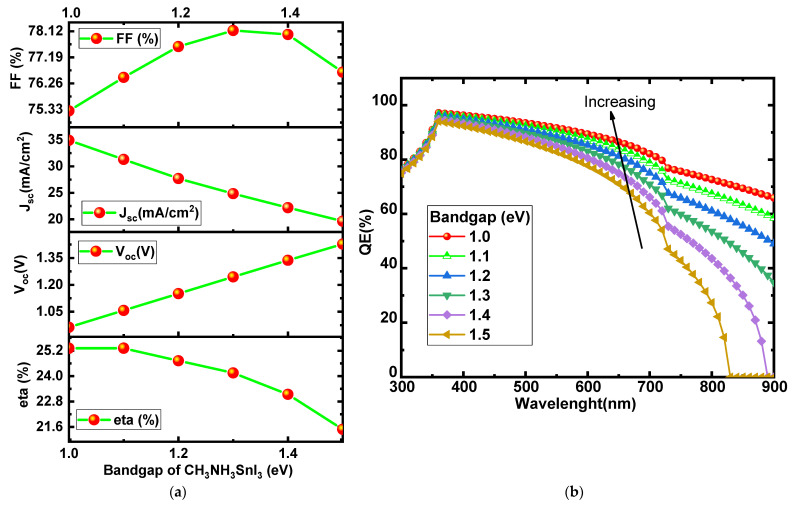
(**a**) Effect of bandgap of perovskite on solar cell parameters (**b**) QE vs. wavelength.

In this present section, the effect of the bandgap on the solar cell performance has been investigated, which is shown in Figure 4a. The energy bandgap of the CH_3_NH_3_SnI_3_ has been varied from 1.0 eV to 1.5 eV and the corresponding change in the performance is noted. FF and Voc increase with the bandgap of absorber materials while Jsc and PCE decrease with an increasing bandgap of absorber materials. The FF and open circuit voltage is proportional to the active material’s bandgap. As the bandgap widens, so does the open circuit voltage [44].

In Figure 4a the sharp decrease in PCE and J_sc_ was observed with the increase in the bandgap of the absorber layer. Initially, FF increases with the bandgap of the absorber layer to the particular bandgap of 1.3 eV, but with further increase, it starts to decrease.

The QE spectra of varying the bandgap of the CH_3_NH_3_SnI_3_ are shown in Figure 4b. QE vs. wavelength curve demonstrates that the quantum efficiency continues to increase with increasing the bandgap of the materials. Initially, maximum QE is observed at a bandgap of 1.0 eV, while with the increase in the bandgap of the absorber layer, QE goes on decreasing, which results in a decrease in the PCE and J_sc_ of the perovskite device.

### 3.3. Effect of Doping Density of ETL

**Figure 5:**
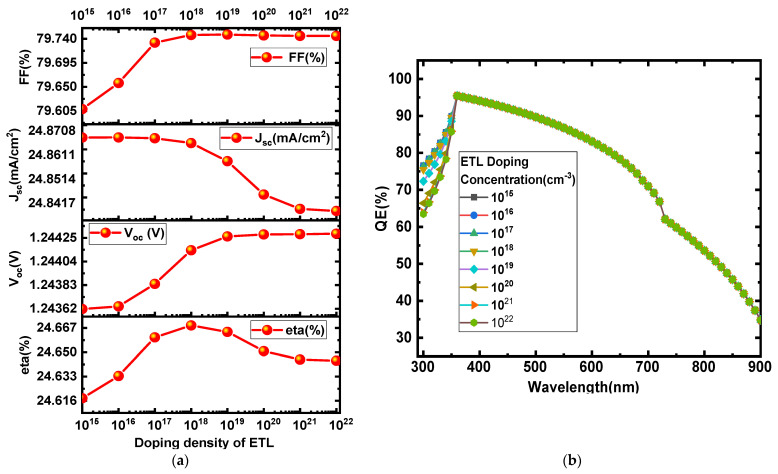
(**a**) Effect of doping density of the ETL on solar cell parameters (J_sc_, V_oc_, FF, and η] (**b**) QE vs. wavelength.

The impact of the doping density of the WS_2_ ETL on the functional parameters of perovskite cells has been investigated. The doping density of the ETL layer has been varied from 10^15^ cm^−3^ to 10^22^ cm^−3^, as shown in Figure 5a. Initially, with increases in the doping density of the ETL material, PCE, V_oc_, and FF go on increasing to a certain value of 10^18^ cm^−3^ and with a further increase in the doping density, V_oc_ and FF become saturated while PCE slightly decreases and then becomes constant.

The QE vs. wavelength curve shows almost constant variation in quantum efficiency with an increase in the doping concentration of the electron transport material (ETM) (Figure 5b). The QE with varied doping density at a wavelength range of ~400–900 nm is observed to be constant, while a slight change can be seen below 400 nm, as shown in Figure 5b.

The doping concentration of the HTL has little impact on Jsc because photogeneration occurs mostly in the absorber layer [45].

The maximum performance of the proposed device structure Glass/ITO/WS_2_/CH_3_NH_3_SnI_3_/P3HT/Au is observed at a doping density of 10^18^ cm^−3^. Therefore, 10^18^ cm^−3^ is taken as the optimum doping density for WS_2_ of this device structure.

### 3.4. Effect of Doping Density and Bandgap of HTL

**Figure 6:**
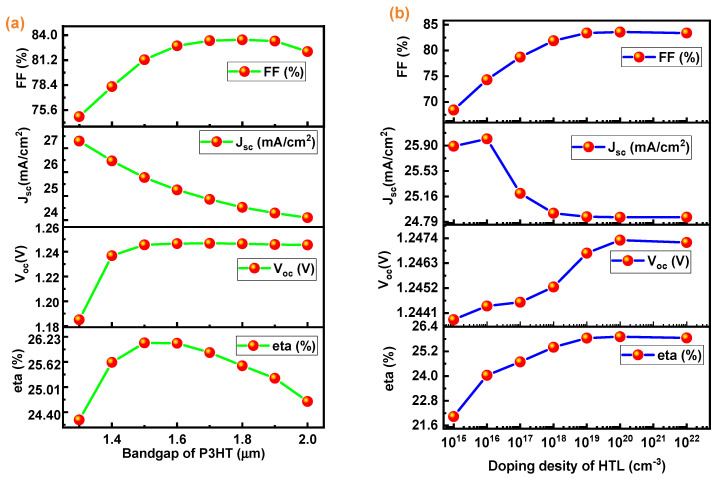
(**a**) Effect of bandgap of P3HT on solar cell parameters (J_sc_, V_oc_, FF, and η] (**b**) Effect of the HTL doping density on the parameters of solar cell (Jsc, Voc, FF, and η).

The effect of the bandgap of the HTL on the performance of the perovskite solar cell has been investigated, which is shown in Figure 6a. Increases in FF, Voc, and PCE are shown with an increase in the HTL’s bandgap, however a substantial decrease in J_sc_ (mA/cm^2^) is seen. Because of the large bandgap, more energy is required to transfer an electron from the valence band to the conduction band.

Furthermore, the influence of the P3HT’s doping density on the perovskite cell’s functional characteristics has been studied. The doping density of the HTL layer has been varied from 10^15^ cm^−3^ to 10^22^ cm^−3^, as shown in Figure 6b. Initially, as the doping density of the ETL material increases, PCE, Voc, and FF increase to a certain threshold prior to getting saturated. While a small drop in J_sc_ (mA/cm^2^) has been noticed with a rise in P3HT doping concentration. Moreover, photogeneration occurs mainly in the Ch_3_NH_3_SnI_3_ layer, and as a result, the doping concentration of the HTL has little effect on J_sc_ [45]_._

From the graph, it is concluded that the maximum performance of the device structure glass/ITO/WS_2_/CH_3_NH_3_SnI_3_/P3HT/Au is at a doping density of 10^18^ cm^−3^. As a result, 10^18^ cm^−3^ is chosen as the best doping density for P3HT in this device configuration.

### 3.5. Optimized Performance

**Figure 7:**
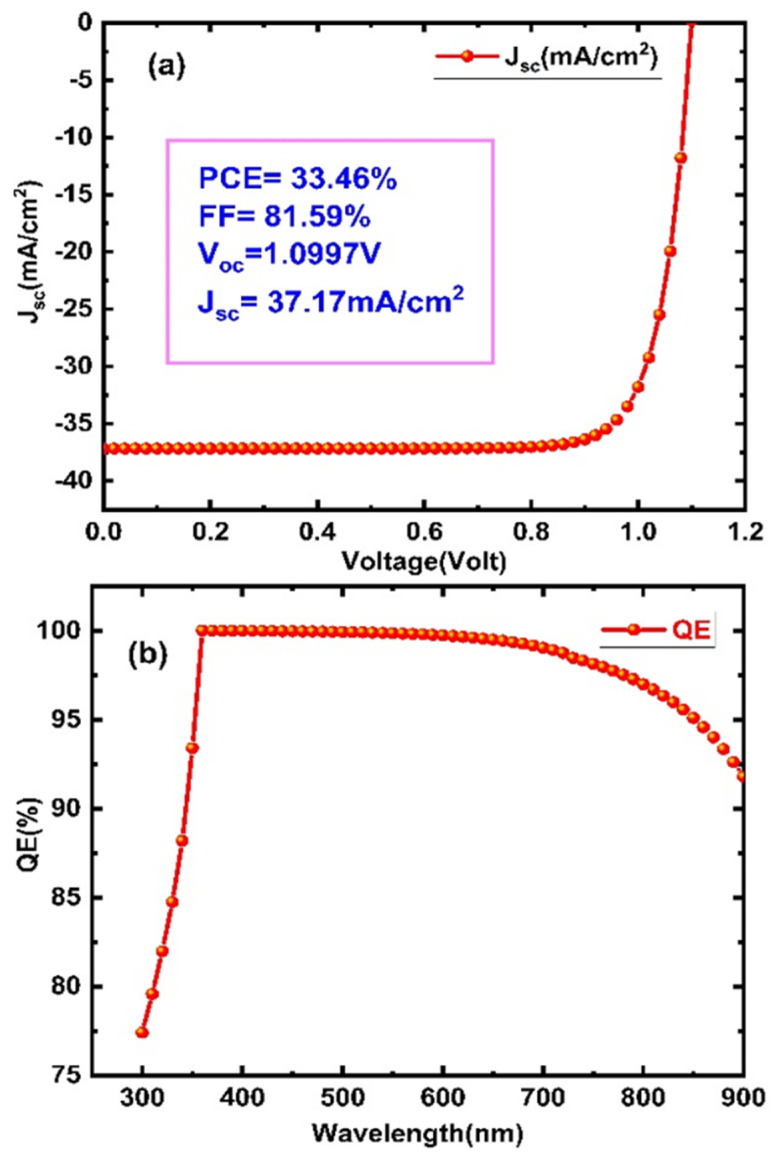
(**a**) Current vs. voltage characteristics and (**b**) QE vs. wavelength of the proposed CH_3_NH_3_SnI_3_ based perovskites solar cell.

The performance of the lead-free perovskite solar cell with the device structure Glass/ITO/WS_2_/CH_3_NH_3_SnI_3_/P3HT have been studied. The optimized performance of the CH_3_NH_3_SnI_3_ perovskite solar cell using WS_2_ as an electron transporting layer is shown in Figure 7a,b. To optimize the performance, different parameters such as thickness, doping concentration, and bandgap have been varied. In this study, room temperature (300 K) is used for optimization. The best PCE with optimized parameters has been observed as 33.36%.

The light transmittance of the substrate, which is known to be over 90% for glass substrates, has a substantial impact on the efficiency of perovskite solar cells. Solar cell power is primarily affected by optical losses via a reduction in the short-circuit current. Light that could have produced an electron-hole pair but does not because it is reflected off the front surface or because it is not absorbed by the solar cell is referred to as an optical loss. The ideal situation would be for visible light (350–780 nm) to be totally absorbed, since it has enough energy to produce electron–hole pairs [46,47]. There are a number of measures to lower optical losses, including coating the top surface of the cell with anti-reflective materials, reducing top contact coverage of the cell surface, increasing absorption by thickening the active layer, reducing reflection by texturing the surface, increasing the optical path by combining surface texturing, and light trapping, among others [48,49].

The potential of these materials, such as WS_2_ and P3HT, to act as electron and hole transport layers in thin film solar cells could be one of the reasons for the high-power conversion efficiency [20,21,22]. Another reason could be optical effects caused by multi-reflection within the device between layers, which could result in the active layer absorbing more photons and producing larger short-circuit currents [50].

The simulated and experimental reported results are listed in Table 2. Our simulated study, according to the tabulated results, is in good agreement with experimentally reported work by various researchers working in this field.

## 4. Conclusions

In the present study, a novel device structure composed of Glass/ITO/WS_2_/CH_3_NH_3_SnI_3_/P3HT was investigated to boost solar cell performance. The lead-free perovskite solar cell can have good efficiency by selecting a buffer layer of wide bandgap materials that can transmit more photons to the absorber layer. Here, the impact of the various parameters on the performance of the perovskite solar cell has been studied. The optimized performance of the proposed device structure such as V_oc_, FF, J_sc_, and PCE are 1.0997 V, 37.1778 mA, 81.59%, and 33.36% respectively. It can also be concluded that with the increase in the ETL doping concentration, the suggested structure has good performance and can compete with the existing lead-based perovskite solar cells. The present study would serve as a beneficial roadmap in developing lead-free, high-efficiency perovskite solar cells.

## Figures and Tables

**Table 1:** Simulation parameters of each layer of the proposed device structure.

Parameters	P3HT [28]	CH_3_NH_3_SnI_3_ [18]	WS_2_ [38]	ITO
Thickness (nm)	350	350	150	100
E_g_ (eV)	1.700	1.3	1.800	3.500
X (eV)	3.500	4.17	3.950	4.000
ε_r_	3.000	8.2	13.600	9.000
N_c_ (1/cm^3^)	2.0 × 10^18^	1 × 10^18^	2.2 × 10^17^	2.2 × 10^18^
N_v_ (1/cm^3^)	2.0 × 10^19^	1 × 10^18^	2.2 × 10^16^	1.8 × 10^18^
V_e_ (cm/s)	1.0 × 10^7^	1.0 × 10^7^	1.0 × 10^7^	1.0 × 10^7^
V_h_ (cm/s)	1.0 × 10^7^	1.0 × 10^7^	1.0 × 10^7^	1.0 × 10^7^
µ_e_ (cm^2^/Vs)	1.8 × 10^−3^	1.6	1.0 × 10^2^	2.0
µ_h_ (cm^2^/Vs)	1.8 × 10^−2^	1.6	1.0 × 10^2^	1.0
N_D_ (1/cm^3^)	-	1.0 × 10^17^	1.0 × 10^18^	2.0 × 10^19^
N_A_ (1/cm^3^)	1.0 × 10^19^	1.0 × 10^17^	-	-

**Table 2:** Comparison with the finding of the simulated and experimentally reported results.

Device Structure	PCE (%)	FF (%)	V_oc_ (Volt)	J_sc_ (mA/cm^2^)	References
Zn_0.75_Mg_0.25_O/CH_3_NH_3_SnI_3_/MASnBr_3_ (simulated)	26.33	82.01	0.95	33.85	[18]
ITO/PEDOT: PSS/CH_3_NH_3_SnI_3_/C_60_/ BCP/Ag (experimental)	17.1%	76.41	1.00	22.95	[51]
FTO/TiO2/Perovskite/ SpiroMeOTAD/Ag(experimental)	15.1	64.2	0.99	23.71	[52]
ITO/PEDOT: PSS/CH_3_NH_3_PbI_3−x_Cl_x_/C_60_/ BCP/Ag (experimental)	21.1	80.3	1.09	22.3	[53]
FTO/TiO_2_/FASnI_3_/Spiro-OMeTAD/Au (simulated)	19.08	33.72	1.18	31.20	[54]
FTO/ TiO_2_-ZnS/Spiro-OMeTAD/Au	14.90%	74.43	1.02	19.07	[46]
WS_2_/CH_3_NH_3_SnI_3_/P3HT (simulated)	33.46	81.59	1.0997	37.17	This work
